# Dendritic cell maturation is induced by *p53*-armed oncolytic adenovirus via tumor-derived exosomes enhancing systemic antitumor immunity

**DOI:** 10.1007/s00262-024-03849-5

**Published:** 2024-11-05

**Authors:** Tomoko Ohtani, Shinji Kuroda, Nobuhiko Kanaya, Yoshihiko Kakiuchi, Kento Kumon, Masashi Hashimoto, Chiaki Yagi, Ryoma Sugimoto, Satoru Kikuchi, Shunsuke Kagawa, Hiroshi Tazawa, Yasuo Urata, Toshiyoshi Fujiwara

**Affiliations:** 1 Department of Gastroenterological Surgery, Okayama University Graduate School of Medicine, Dentistry and Pharmaceutical Sciences, 2-5-1 Shikata-cho, Kita-ku, Okayama, 700-8558 Japan; 2 Minimally Invasive Therapy Center, Okayama University Hospital, Okayama, Japan; 3 Center for Clinical Oncology, Okayama University Hospital, Okayama, Japan; 4 Center for Innovative Clinical Medicine, Okayama University Hospital, Okayama, Japan; 5 Oncolys BioPharma, Inc, Tokyo, Japan

**Keywords:** Oncolytic adenovirus, *p53*, Dendritic cells, Anti-tumor immunity, Exosome

## Abstract

Dendritic cells (DCs) are crucial in cancer immunity, because they activate cytotoxic T cells by presenting tumor antigens. Recently, oncolytic virus therapy has been recognized as a systemic immune stimulator. We previously developed a telomerase-specific oncolytic adenovirus (OBP-301) and a p53-armed OBP-301 (OBP-702), demonstrating that these viruses strongly activate systemic antitumor immunity. However, their effects on DCs remained unclear. In the present study, the aim was to elucidate the mechanisms of DC activation by OBP-702, focusing particularly on tumor-derived exosomes. Exosomes (Exo53, Exo301, or Exo702) were isolated from conditioned media of human or murine pancreatic cancer cell lines (Panc-1, MiaPaCa-2, and PAN02) after treatment with Ad-p53, OBP-301, or OBP-702. Exo702 derived from Panc-1 and MiaPaCa-2 cells significantly upregulated CD86, CD80, CD83 (markers of DC maturation), and IFN-γ in DCs in vitro. Similarly, Exo702 derived from PAN02 cells upregulated CD86 and IFN-γ in bone marrow-derived DCs in a bilateral PAN02 subcutaneous tumor model. This DC maturation was inhibited by GW4869, an inhibitor of exosome release, and anti-CD63, an antibody targeting the exosome marker. Intratumoral injection of OBP-702 into PAN02 subcutaneous tumors significantly increased the presence of mature DCs and CD8-positive T cells in draining lymph nodes, leading to long-lasting antitumor effects through the durable activation of systemic antitumor immunity. In conclusion, tumor-derived exosomes play a significant role in DC maturation following OBP-702 treatment and are critical for the systemic activation of antitumor immunity, leading to the abscopal effect.

**Supplementary Information:**

The online version contains supplementary material available at 10.1007/s00262-024-03849-5.

## Introduction

Dendritic cells (DCs), first reported by Steinman in 1973, are important professional antigen-presenting cells (APCs) that play a critical role in both innate and adaptive immune responses [1]. DCs are one of the main components of the cancer-immunity cycle and activate cytotoxic T cells by cross-presenting tumor-associated antigens (TAAs), which are processed into peptides and presented on major histocompatibility complex (MHC) molecules, to naïve T cells [2, 3]. Mature DCs express high levels of MHC class I and II, the co-stimulatory molecules CD80 and CD86, which provide the signal required for T cell activation via interaction with CD28, and secrete cytokines such as interleukin (IL)-12 and interferon (IFN)-γ. As DC-based immunotherapy has developed over the decades, activation of DCs leading to cytotoxic T cell activation is still considered an intriguing strategy in cancer immunotherapy.

Oncolytic virus therapy is an emerging novel therapeutic class and highly anticipated as a systemic immune stimulator [4]. The superiority of oncolytic virus-based immunotherapy relative to other approaches lies in its tumor specificity [5]. Some oncolytic viruses (OVs) such as talimogene laherparepvec (T-VEC) have already been in clinical use, and many other OVs have been tested in clinical trials [6], one of which is a telomerase-specific oncolytic adenovirus named suratadenoturev (OBP-301) that we previously developed [7–10]. We showed that OBP-301 treatment stimulated DCs via producing the endogenous danger signal, uric acid [11]. Then, we developed another telomerase-specific oncolytic adenovirus armed with p53 tumor suppressor gene (OBP-702) as a next-generation agent [12]. In fact, OBP-702 showed stronger antitumor activity than OBP-301 even against refractory cancers such as pancreatic cancer by activation of systemic antitumor immunity, in addition to oncolytic cell death and p53-mediated cytotoxic effects [13–16]. In addition, p53, known as “the guardian of the genome”, regulates cell division and prevents tumor formation by controlling the cell cycle, DNA repair machinery, and metabolism, and p53 replacement therapy has shown potent anti-tumor effects in preclinical and clinical studies [17–19]. Furthermore, p53 also interacts with Wnt/β-catenin signaling, which prevents T cell migration into tumor tissues mediated by DCs, and modulation of the Wnt/β-catenin signaling pathway via p53 is considered an attractive therapeutic target [20–22].

Exosomes are now recognized as one form of extracellular vesicle, along with ectosomes and apoptotic bodies, differentiated based on origin and size, that play important roles in various intracellular communication processes such as immune responses and delivery systems [23, 24]. Exosomes contain proteins, lipids, and nucleic acids derived from their host cells, and tumor-derived exosomes are involved in the establishment of the tumor microenvironment and the occurrence of metastasis [25, 26]. We previously reported that OBP-301 or OBP-301-related components such as DNA and proteins were contained in exosomes secreted from tumor cells after OBP-301 treatment, and these tumor-derived exosomes secreted following OBP-301 treatment played an important role in the abscopal effect of OBP-301 through the direct delivery of OBP-301 or OBP-301-related components to the metastatic site [27].

Though we have shown that local treatment of oncolytic adenoviruses strongly induced immunogenic cell death (ICD) in tumor tissues, activated systemic antitumor immunity, and caused antitumor effects by recruitment of cytotoxic T cells into tumor tissues, the effects on DCs in this antitumor activity by oncolytic adenoviruses remained unclear. In the present study, the aim was to uncover the precise mechanisms for activation of antitumor immunity by p53-armed oncolytic adenovirus OBP-702, focusing especially on the effects on DCs via tumor-derived exosomes secreted following OBP-702 treatment.

## Materials and methods

### Cell lines and cell cultures

The human pancreatic ductal adenocarcinoma (PDAC) cell lines Panc-1 and MiaPaCa-2 were purchased from the American Type Culture Collection (ATCC, Manassas, VA, USA) and cultured in DMEM medium supplemented with 10% fetal bovine serum (FBS) and 1% penicillin-streptomycin (100 U/ml). The murine PDAC cell line PAN02 derived from C57BL/6 mice was purchased from the National Cancer Institute (Frederick, MD, USA) and cultured in RPMI medium supplemented with 10% FBS and 1% penicillin-streptomycin (100 U/ml). The human monocytic leukemia cell line THP-1 was purchased from the ATCC and cultured in RPMI medium supplemented with 10% FBS and 1% penicillin streptomycin (100 U/ml). Neither cell line was cultured for more than 3 months following resuscitation. Cell authentication was not performed by the authors.

### Oncolytic adenoviruses

Ad/CMV-*p53* (Ad-p53), suratadenoturev (OBP-301), and OBP-702 were used in this study (Fig. S1). Ad-p53 is a replication-deficient recombinant adenovirus containing cytomegalovirus (CMV) promoter driving *p53* gene. The telomerase-specific replication-competent adenovirus OBP-301, in which the promoter element of the *hTERT* gene drives the expression of *E1A* and *E1B* genes, was constructed and characterized previously [7, 28, 29]. OBP-702 was constructed by modifying OBP-301 to express the exogenous *p53* gene by inserting a human wild-type *p53* gene expression cassette driven by the Egr-1 promoter into the E3 region of OBP-301 [12]. Viruses were stored at − 80 °C.

### Isolation and characterization of exosomes

Normal exosomes were isolated by ultracentrifugation of supernatants collected after cell culture in FBS-free medium for 48 h (Exo). Exosomes were also isolated by the same process following Ad-p53, OBP-301, or OBP-702 treatment at the IC80 dose of OBP-702 for 24 h (Exo53, Exo301, or Exo702). Briefly, the ultracentrifugation method involved centrifuging the collected supernatants at 2,000 × g for 10 min to remove cells and debris, followed by another centrifugation at 100,000 × g for 70 min at 4 °C after filtration through a 0.22-µm filter. The pellets were then rinsed with phosphate-buffered saline (PBS), ultra-centrifuged again with the same conditions, and dispersed in PBS. Protein concentrations were measured using the bicinchoninic acid (BCA) assay according to the manufacturer’s protocol (Thermo Fisher, Waltham, MA, USA). The sizes of exosomes were measured using dynamic light scattering (DLS) with a Zetasizer Nano ZSP (Malvern Instruments, Malvern, UK). The morphology and structure of exosomes were visualized using a transmission electron microscope (TEM) (H-7560, HITACHI, Hitachi, Japan).

### Cell viability assay

Panc-1, MiaPaCa-2, and PAN02 cells were seeded in 96-well plates (2 × 10^3^ cells/well) (*n* = 5) and treated with OBP-702, OBP-301, or Ad-p53 at the indicated multiplicity of infection (MOI) or treated with various concentrations of Exo, Exo53, Exo301, or Exo702. Cell viability was determined 3 days after treatment using a Cell Proliferation Kit II (XTT) (Roche Diagnostics) according to the manufacturer’s protocol.

### Western blot analysis

Proteins extracted from whole-cell lysates were electrophoresed on 10–15% SDS-polyacrylamide gels and transferred onto Hybond-polyvinylidene difluoride transfer membranes (GE Healthcare UK Ltd., Buckinghamshire, UK). The membranes were incubated with primary antibodies against E1A (M58, BD Biosciences, Franklin Lakes, NJ, USA), Adenovirus 5 (ab6982, abcam, Cambridge, UK), p53 (DO-1, Invitrogen, Waltham, MA, USA), poly(ADP ribose) polymerase (PARP) (cat 9542, Cell Signaling Technology, Danvers, MA, USA), p62 (SQSTM1) (cat 5114, Cell Signaling Technology), Wnt16 (H-96, Santa Cruz, Dallas, TX, USA), β-catenin (D10A8, Cell Signaling Technology), CD9 (Ts9, Thermo Fisher), CD63 (Ts63, Thermo Fisher), CD81 (M38, Thermo Fisher), and β-actin (AC-15, Sigma-Aldrich, Saint Louis, MO, USA), followed by peroxidase-linked secondary antibody. The Amersham ECL chemiluminescence system (GE Healthcare UK Ltd.) was used to detect the peroxidase activity of the bound antibody. Equal loading of samples was confirmed using β-Actin.

### ATP assay

Panc-1 and MiaPaCa-2 cells were treated with Ad-p53, OBP-301, or OBP-702 at the IC80 dose of OBP-702 for 72 h (*n* = 3), and the levels of extracellular ATP in the supernatants were measured using an ENLITEN ATP assay (Promega, Madison, WI, USA) according to the manufacturer’s protocols.

### Generation of dcs

The differentiation of THP-1 cells into immature DCs was achieved by culturing the cells in the presence of recombinant human IL-4 (100 ng/mL) (Sigma-Aldrich) and GM-CSF (100 ng/mL) (Sigma-Aldrich) for five days. Medium exchange was performed every 2 days with fresh cytokine-supplemented medium. For the stimulation of immature DCs, cells were incubated in serum-free medium for 48 h with exosomes isolated from Panc-1 cells and used for further tests.

DCs were also differentiated from bone marrow of C57BL/6 mice. Bone marrow harvested from 6-week-ols C57BL/6 mice was cultured in RPMI-1640 medium in the presence of rmGM-CSF (40 ng/ml) (Sigma-Aldrich) and rmIL-4 (10 ng/ml) (Sigma-Aldrich). After 5 days, loosely attached immature DCs were stimulated with exosomes isolated from PAN02 cells and used for further tests.

### Flow cytometry

Flow cytometry for DC surface markers (CD80 [2D10, Biolegend, San Diego, CA, USA], CD83 [HB15e, Biolegend], CD86 [cat 772906, Biolegend]) and intracellular cytokine (IFN-γ [4 S.B3, Biolegend]) was performed using FACS Array and BD FACS Aria (BD Biosciences, San Jose, CA, USA), and analysis was performed using FlowJo software (TreeStar). For intracellular staining of IFN-γ, cells were treated with GolgiStop (BD Biosciences) for 4 h prior to staining. Cells were permeabilized for 30 min and stained for intracellular proteins for 1 h at room temperature.

For the in vivo experiments, cells were incubated with Zombie aqua fixable viability kit (Biolegend) and Fc Block (Biolegend) for 20 min on ice. Single cell suspensions were stained for surface markers (CD86 [GL-1, Biolegend], CD11b [M1/70, Biolegend], CD11c [N418, Biolegend], MHC-II [M5/114.15.2, Biolegend]) in PBS for 15 min at room temperature. For intracellular staining of IFN-γ (XMG1.2, Biolegend), cells were treated with GolgiStop (BD Biosciences) for 4 h prior to staining.

### Reagents

The ExoCap Streptavidin CD9/CD63/CD81 Set (cat. MEX-SA123; MBL International, Woburn, MA, USA) was used to extract pure exosomes. GW4869 (Sigma-Aldrich) was used to inhibit exosome secretion. Anti-CD63 antibody (1:1000, cat. 10628D, Invitrogen) was used to inhibit exosome function.

### Cytokine assay

CXCL10 secreted from DCs derived from THP1 cells (4 × 10^5^ cells/6-well plate) was measured using the CXCL10 ELISA kit (R&D Systems, Minneapolis, MN, USA) according to the manufacturer’s protocols.

### In vivo experiments

PAN02 cells (2 × 10^6^ cells) were subcutaneously injected into the bilateral flanks of 6-week-old female C57BL/6 mice. Treatment was initiated when tumors reached a diameter of approximately 5 mm. The perpendicular diameter of each tumor was measured 3 times a week, and tumor volume was calculated using the following formula: tumor volume (mm^3^) = *a*×*b*^*2*^ × 0.5, where *a* represents the longest diameter, *b* represents the shortest diameter, and 0.5 is a constant used to calculate the volume of an ellipsoid.

One side was treated with Exo702 (20 µg/body) or PBS intratumorally 3 times a week, and the other side was left untreated. Mice were sacrificed 7 days after treatment initiation to check the effects on draining lymph nodes and tumor-infiltrating lymphocytes (TILs).

One side was treated with Ad-p53, OBP-301, OBP-702 (1 × 10^9^ plaque-forming units [PFU]), or PBS intratumorally 3 times a week, and the other side was left untreated. The mice were sacrificed 7 days after treatment initiation to check the effects on draining lymph nodes and TILs.

One side was treated with Ad-p53, OBP-301, OBP-702 (1 × 10^9^ PFU), or PBS intratumorally 3 times a week, and the other side was left untreated. Tumor volume was monitored until 28 days after treatment initiation to assess the therapeutic efficacy of OBP-702, and the mice were sacrificed to investigate the effects on TILs.

Mice were housed in a specific pathogen-free environment in the Department of Animal Resources of Okayama University. All animal experimental protocols were approved by the Institutional Animal Care and Use Committee of Okayama University.

### Immunohistochemistry

Formalin-fixed, paraffin-embedded tissue samples cut at 4 μm were deparaffinized in xylene and rehydrated using a graded ethanol series. After blocking endogenous peroxidases by incubation with 3% H_2_O_2_ for 10 min, the samples were boiled in citrate buffer or EDTA buffer for 14 min in a microwave oven for antigen retrieval. The samples were incubated with primary antibodies overnight at 4 °C and then with peroxidase-linked secondary antibody for 30 min at room temperature. Samples were stained with 3,3-diaminobenzidine for signal generation, counterstained with Mayer’s hematoxylin, and then dehydrated and mounted onto coverslips. Antibodies to CD11c (1:350, D1V9Y, Cell Signaling Technology), CD8a (1:200, 4SM15, Thermo Fisher), and p53 (7F5) (1:300, cat. 2527; Cell Signaling Technology) were used. The specimens were then photographed and analyzed using Olympus cellSens software.

### CTL assay

PAN02 cells (2 × 10^6^ cells) were subcutaneously injected into the flanks of 6-week-old female C57BL/6 mice. PAN02 subcutaneous tumors were treated intratumorally with PBS, Ad-p53, OBP-301, or OBP-702 injection (1 × 10^8^ PFU) 3 times a week, and the mice were sacrificed 7 days after treatment initiation (*n* = 3). Splenocytes collected by homogenization of the spleen were treated by red blood cell (RBC) lysis buffer (BioLegend) and debris removal solution kit (Miltenyi Biotec, Bergisch Gladbach, Germany). The splenocytes were then co-incubated with PAN02 cells, which were irradiated with 100 Gy and incubated with IFN-γ (100 U/mL) (Wako Pure Chemical Industries, Osaka, Japan) for 5 days for stimulation. PAN02 cells were incubated with the stimulated splenocytes for 4 h at 37 °C at a ratio of 1:62.5. Cytotoxicity was evaluated with the Cytotoxicity Detection Kit plus (LDH) (Sigma Aldrich) according to the manufacturer’s protocols.

### Statistical analysis

Statistical analysis was performed using JMP software (SAS Institute, Cary, NC, USA). Student’s *t*-test was used to assess the significance of differences in most continuous variables, except for quantitative immunohistochemical analyses of CD11c, CD8, and p53, for which the Wilcoxon signed-rank test was used. *P* values < 0.05 were considered significant.

## Results

### Cytotoxic activity of OBP-702 against PDAC cell lines

**Fig. 1:**
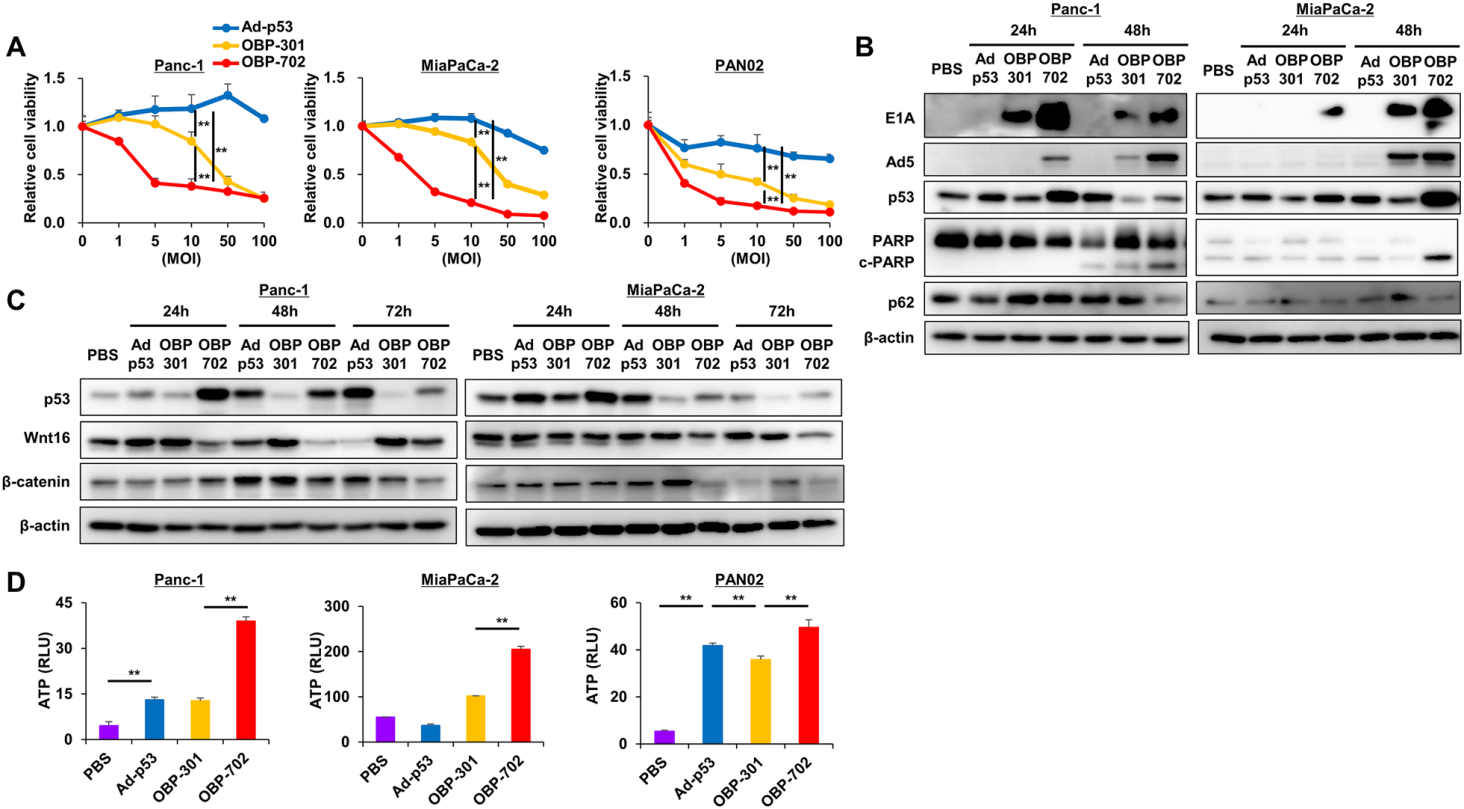
Cytotoxic activity of OBP-702 against PDAC cell lines **A** Viability of Panc-1, MiaPaCa-2, and PAN02 cells was assessed using an XTT assay 3 days after treatment with Ad-p53, OBP-301, or OBP-702 at the indicated doses (MOI) (*n* = 5). The percentage of viable cells relative to non-treated cells (0 MOI) was plotted. Error bars indicate 95% confidence intervals. **, *p* < 0.01 (Student’s *t*-test). **B** Whole-cell lysates of Panc-1 and MiaPaCa-2 cells collected 24 and 48 h after Ad-p53, OBP-301, or OBP-702 treatment (MOI of IC50) were subjected to western blot analysis of E1A, Adenovirus5 (Ad5), p53, PARP, cleaved-PARP (c-PARP), p62, and β-Actin. **C** Whole-cell lysates of Panc-1 and MiaPaCa-2 cells collected 24, 48, and 72 h after Ad-p53, OBP-301, or OBP-702 treatment (MOI of IC50) were subjected to western blot analysis of p53, Wnt16, β-catenin, and β-actin. **D** Levels of extracellular ATP secreted from Panc-1, MiaPaCa-2, and PAN02 cells were measured using a luminescence assay 72 h after Ad-p53, OBP-301, or OBP-702 treatment (MOI of IC50) (*n* = 3). **, *p* < 0.01 (Student’s *t*-test)

OBP-702 showed dose-dependent cytotoxicity on Panc-1, MiaPaCa-2, and PAN02 cells, which was significantly stronger than Ad-p53 and OBP-301 at most doses including 10 MOI, at which there was significant differences between OBP-702 and Ad-p53 or OBP-301 (Fig. 1A). This cytotoxicity of OBP-702 was due to induction of apoptosis and autophagy, shown by upregulation of cleaved PARP and downregulation of p62, respectively, following induction of adenoviral E1A and p53, which was evident 48 h after treatment and stronger than that of Ad-p53 and OBP-301 (Fig. 1B). Wnt/β-catenin expressions were strongly reduced following p53 induction after OBP-702 treatment of Panc-1 and MiaPaCa-2 cells (Fig. 1C). OBP-702 induced significantly greater ATP release from Panc-1, MiaPaCa-2, and PAN02 cells 72 h after treatment than Ad-p53 and OBP-301 (Fig. 1D).

### Characteristics of PDAC cell-derived exosomes released by OBP-702 treatment

**Fig. 2:**
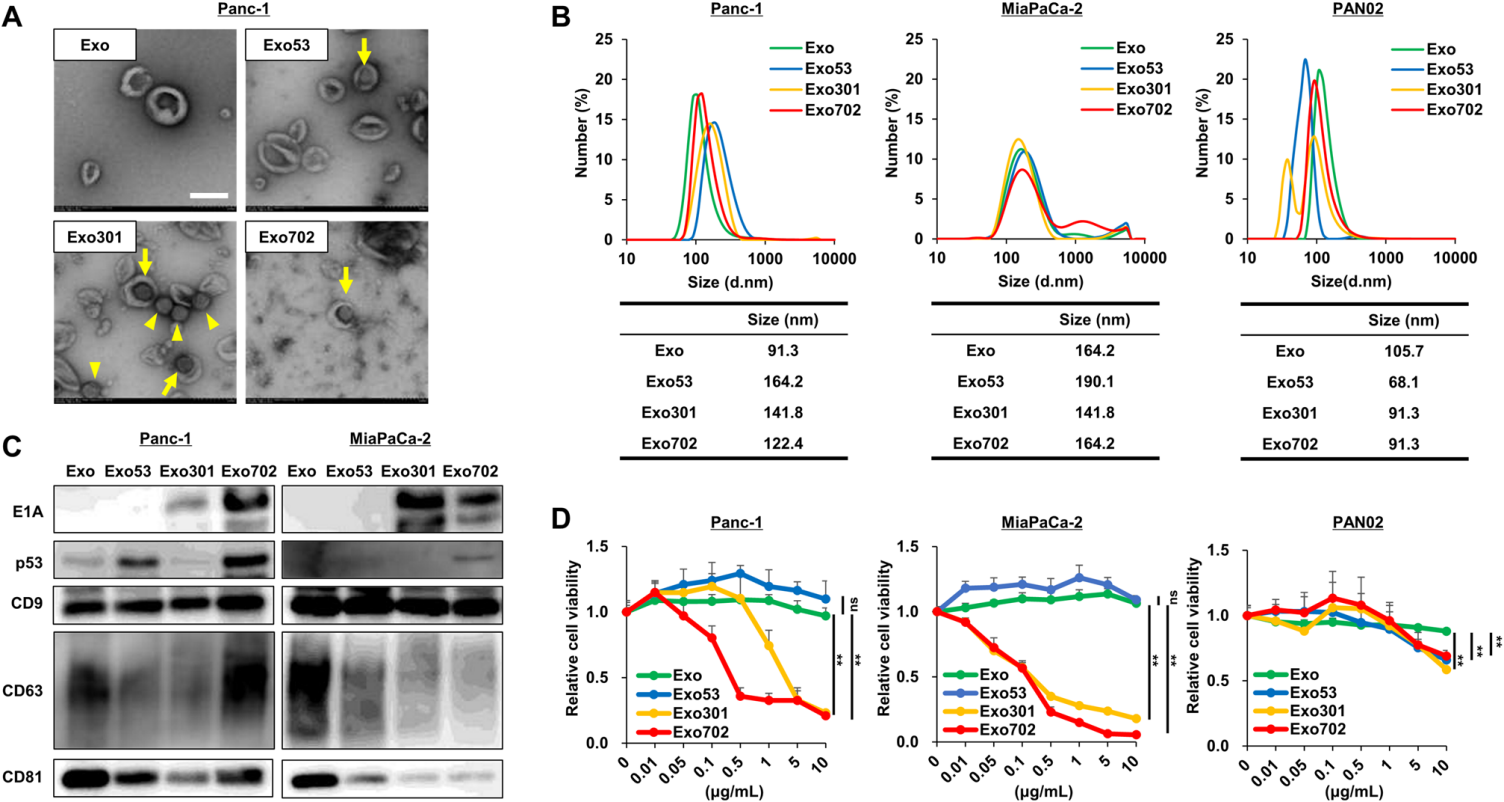
Characteristics of PDAC cell-derived exosomes released by OBP-702 treatment **A** Representative TEM images of exosomes isolated from Panc-1 cells treated with PBS (Exo), Ad-p53 (Exo53), OBP-301 (Exo301), or OBP-702 (Exo702). Scale bar, 200 nm. Arrows indicate adenovirus inside exosomes. Arrowheads indicate adenovirus in free space. **B** Particle sizes of Exo, Exo53, Exo301, and Exo702 derived from Panc-1, MiaPaCa-2, and PAN02 cells were measured by DLS. The size of the peak wavelength of each exosome is shown in the table. **C** Lysates of Exo, Exo53, Exo301, and Exo702 derived from Panc-1 and MiaPaCa-2 cells were subjected to western blot analysis of E1A, p53, CD9, CD63, and CD81. **D** Viability of Panc-1, MiaPaCa-2, and PAN02 cells was assessed using an XTT assay 3 days after treatment with Exo, Exo53, Exo301, or Exo702 at the indicated concentrations (*n* = 5). The percentage of viable cells relative to non-treated cells is plotted. Error bars indicate 95% confidence intervals. ns, not significant. **, *p* < 0.01 (Student’s *t*-test)

TEM demonstrated that all exosomes (Exo, Exo53, Exo301, Exo702) derived from Panc-1 cells were spherical in shape, and some adenoviruses were observed inside exosomes, as well as in a free space in Exo301 and Exo702 (Fig. 2A). DLS analysis showed that the size of Exo53, Exo301, and Exo702 derived from Panc-1, MiaPaCa-2, and PAN02 cells was approximately 60–200 nm in diameter (Fig. 2B). Western blot analysis for exosomes showed that adenoviral E1A protein was contained in Exo301 and Exo702, and p53 protein was contained in Exo53 and Exo702, with exosome-related proteins such as CD9, CD63, and CD81 on Panc-1, MiaPaCa-2, and PAN02 cells (Fig. 2C, Fig. S2). In addition, Exo301 and Exo702 showed significant cytotoxic potential on Panc-1, MiaPaCa-2, and PAN02 cells, similar to each original virus of OBP-301 and OBP-702 (Fig. 2D). When ExoCap (EC) was used to extract pure exosomes from Exo (EC-Exo), Exo53 (EC-Exo53), Exo301 (EC-Exo301), and Exo702 (EC-Exo702), EC-Exo301 and EC-Exo702 also showed significant cytotoxic potential on Panc-1 and MiaPaCa-2 cells (Fig. S3). These findings indicated that cancer-derived exosomes released by OV treatment contained the OV component and showed similar cytotoxic potential to OVs themselves.

### Potential effect of exo702 derived from human PDAC cells on DC maturation

**Fig. 3:**
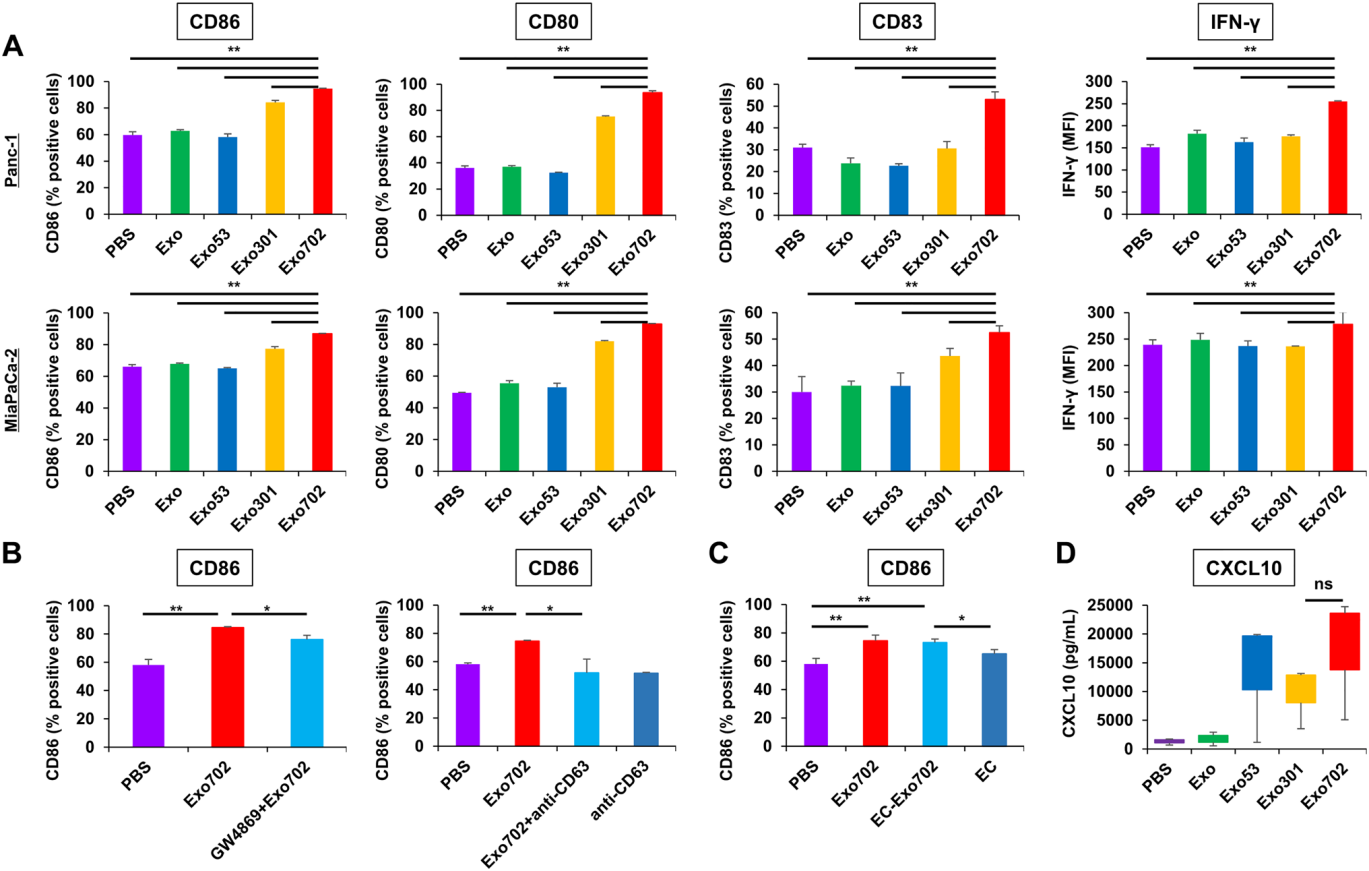
Potential effects of Exo702 derived from human PDAC cells on DC maturation **A** DCs derived from THP-1 cells were subjected to flow cytometry for CD86, CD80, and CD83 expressed on the cell surface and intracellular IFN-γ 2 days after treatment with Exo, Exo53, Exo301, or Exo702 derived from Panc-1 and MiaPaCa-2 cells (*n* = 3). **, *p* < 0.01 (Student’s *t*-test). **B** THP-1-derived DCs were subjected to flow cytometry for CD86 on the cell surface 2 days after treatment with Exo702 or GW4869 + Exo702, and Exo702, anti-CD63, or Exo702 + anti-CD63 derived from Panc-1 cells (*n* = 3). GW4869 was added to the culture medium 6 h prior to OBP-702 treatment to block exosome production. Anti-CD63 was added concurrently with Exo702 to inhibit the function of exosomes. *, *p* < 0.05. **, *p* < 0.01 (Student’s *t*-test). **C** THP-1-derived DCs were subjected to flow cytometry for CD86 on the cell surface 2 days after treatment with Exo702, EC-Exo702, or EC derived from Panc-1 cells (*n* = 3). EC was used to extract pure exosomes. *, *p* < 0.05. **, *p* < 0.01 (Student’s *t*-test). **D** The culture medium of DCs was subjected to ELISA for CXCL10 48 h after treatment with Exo, Exo53, Exo301, or Exo702 derived from Panc-1 cells (*n* = 5). ns, not significant (Wilcoxon signed-rank test)

The potential of Exo702 to stimulate DCs was first examined in an in vitro study using human PDAC cells and THP-1 cells. Exo702 derived from Panc-1 and MiaPaCa-2 cells significantly upregulated the expressions of CD86, CD80, and CD83, markers of DC maturation, and IFN-γ, compared with Exo, Exo53, and Exo301 (Fig. 3A, Fig. S4). The upregulation of CD86 by Exo702 was significantly inhibited by GW4869, an inhibitor of exosome production, and the antibody to CD63, one of the exosome markers (Fig. 3B. Fig. S2C). Since there was a concern that substances not related to exosomes such as free adenoviruses were contaminants in exosomes collected by the ultracentrifugation method, and these might have played a central role in this DC maturation, when ExoCap (EC) was used to extract pure exosomes from Exo702 (EC-Exo702), EC-Exo702 significantly upregulated CD86 expression to a level close to Exo702 (Fig. 3C). Exo702 derived from Panc-1 cells also increased CXCL10/IP-10 secretion from DCs, which is known to play an important role in recruiting TILs, although there was a significant difference between Exo702 and Exo53 or Exo301 (Fig. 3D). These results showed that Exo702, mainly exosome-related substances, was significantly involved in the activation of DCs, leading to recruiting and activating immune cells.

### Potential effects of Exo702 derived from murine PDAC cells on DC maturation and systemic activation of antitumor immunity

**Fig. 4:**
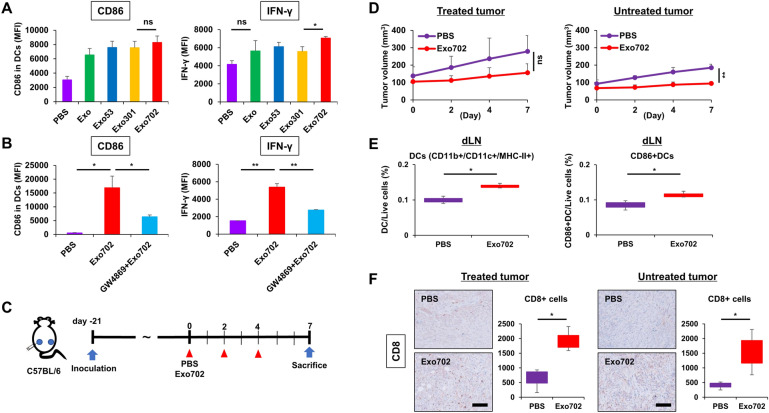
Potential effects of Exo702 derived from murine PDAC cells on DC maturation and systemic activation of antitumor immunity **A** DCs derived from murine bone marrow were subjected to flow cytometry for CD86 on the cell surface and intracellular IFN-γ 2 days after treatment with Exo, Exo53, Exo301, or Exo702 derived from PAN02 cells (*n* = 3). ns, not significant. *, *p* < 0.05 (Student’s *t*-test). **B** Bone marrow-derived DCs were subjected to flow cytometry for CD86 on the cell surface and intracellular IFN-γ 2 days after treatment with Exo702 or GW4869 + Exo702 derived from PAN02 cells (*n* = 3). *, *p* < 0.05. **, *p* < 0.01 (Student’s *t*-test). **C** Study protocol. Briefly, in a bilateral PAN02 subcutaneous tumor model, one side was treated with Exo702 intratumorally (20 µg) 3 times a week, and the other side was left untreated. Mice were sacrificed 7 days after treatment initiation for analysis (*n* = 3). **D** PAN02 tumor volumes of both sides were monitored separately until 7 days after treatment initiation of Exo702 (*n* = 3). ns, not significant. **, *p* < 0.01 (Student’s *t*-test). **E** Draining lymph nodes (dLNs) harvested 7 days after treatment initiation of Exo702 were subjected to flow cytometry for CD11b+/CD11c+/MHC-II+ (DCs) and CD86-positive DCs (*n* = 3). *, *p* < 0.05 (Wilcoxon signed-rank test). **F** Representative figures of immunohistochemical staining for CD8 in treated and untreated tumors harvested 7 days after treatment initiation are shown. Scale bar, 200 μm. Median numbers of CD8-positive cells from 5 selected fields were assessed statistically (*n* = 3). *, *p* < 0.05 (Wilcoxon signed-rank test)

Next, DCs differentiated from mouse bone marrow were used and reacted with exosomes derived from murine PAN02 cells. Exo702 tended to upregulate CD86 expression, although there was no significant difference with Exo301, and significantly increased IFN-γ secretion from DCs (Fig. 4A). Upregulations of CD86 and IFN-γ were significantly inhibited by GW4869, showing that exosomes were involved in this immune modulation (Fig. 4B, Fig. S2B). When the abscopal effect of Exo702 was evaluated using a PAN02 bilateral subcutaneous tumor model (Fig. 4C), intratumoral injection of Exo702 exhibited small therapeutic effects on both the directly treated tumors and the untreated tumors 7 days after treatment initiation, although there was no significant difference in the analysis of relative tumor volume (Fig. 4D, Fig. S5). At the same 7 days after treatment initiation, the recruitment of whole DCs (CD11b + CD11c+/MHC-II+) and matured DCs (CD86+/CD11b+/CD11c+/MHC-II+) into draining lymph nodes was significantly increased by Exo702 intratumoral injection (Fig. 4E, Fig. S6). The recruitment of CD8-positive cells into tumors was also significantly increased by Exo702 injection in both treated and untreated tumors (Fig. 4F). These results showed that Exo702 has the potential to stimulate DCs and activate systemic antitumor immunity in the in vivo mouse model during the very early period when the abscopal effect was barely observed.

### Potential effects of OBP-702 on DC maturation and systemic activation of antitumor immunity in mice bearing PDAC tumors

**Fig. 5:**
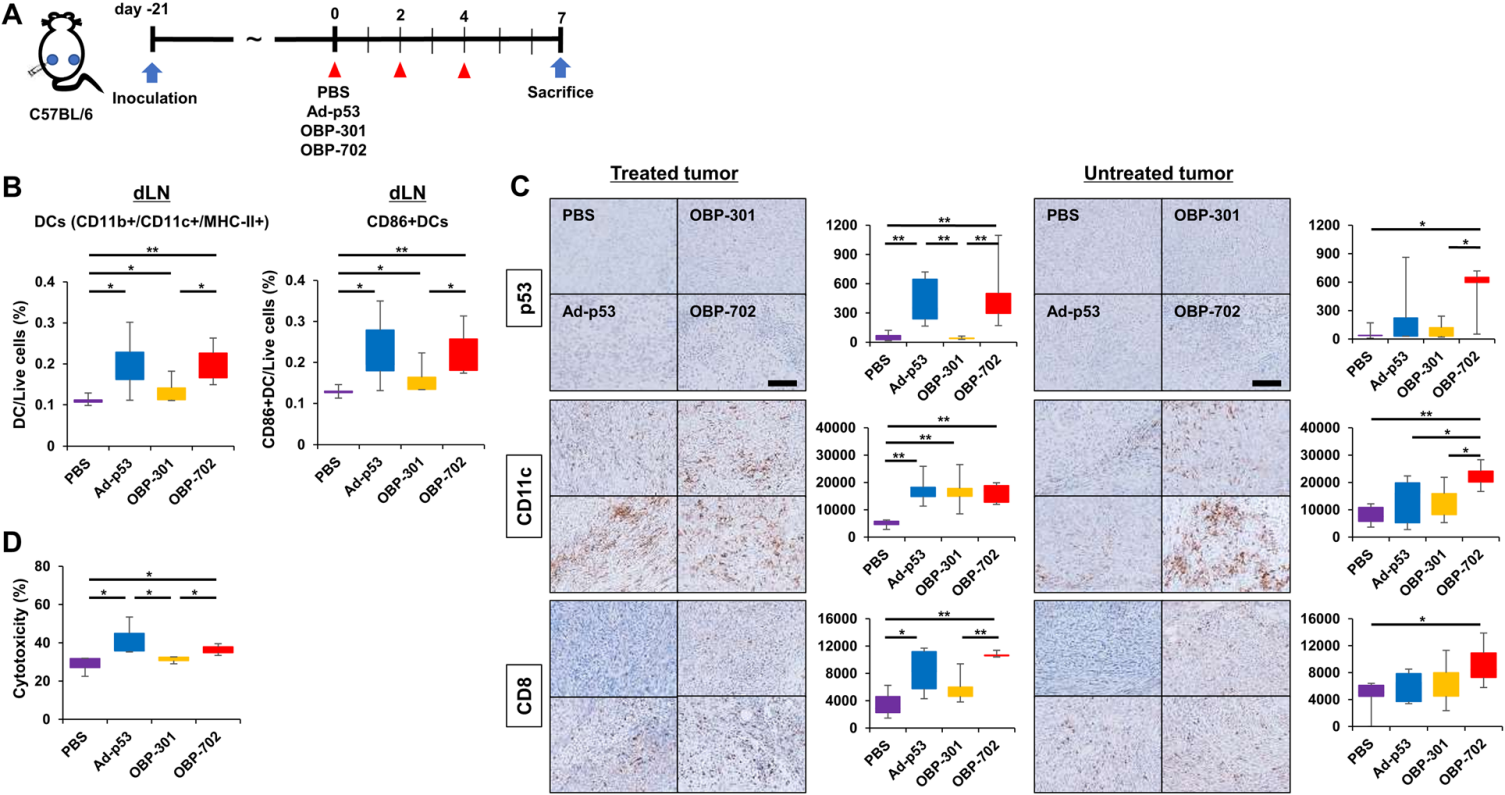
Potential effects of OBP-702 on DC maturation and systemic activation of antitumor immunity in mice bearing PDAC tumors **A**) Study protocol. Briefly, in a bilateral PAN02 subcutaneous tumor model, one side was treated with Ad-p53, OBP-301, or OBP-702 intratumorally (1 × 10^8^ PFU) 3 times a week, and the other side was left untreated. Mice were sacrificed 7 days after treatment initiation for analysis (*n* = 5). **B** Draining lymph nodes (dLNs) harvested 7 days after treatment initiation were subjected to flow cytometry for CD11b+/CD11c+/MHC-II+ (DCs) and CD86-positive DCs (*n* = 5). *, *p* < 0.05. **, *p* < 0.01 (Wilcoxon signed-rank test). **C** Representative figures of immunohistochemical staining for p53, CD11c, and CD8 in treated and untreated tumors harvested 7 days after treatment initiation are shown. Scale bar, 200 μm. Median numbers of p53, CD11c, and CD8-positive cells from 5 selected fields were assessed statistically (*n* = 5). *, *p* < 0.05. **, *p* < 0.01 (Wilcoxon signed-rank test). **D** CTL assay. Cytotoxicity of splenocytes collected from mice treated with Ad-p53, OBP-301, or OBP-702 on PAN02 cells was assessed using the Cytotoxicity Detection Kit plus (LDH) 3 days after treatment (*n* = 3). *, *p* < 0.05 (Wilcoxon signed-rank test)

When the potential of OBP-702 to modulate the antitumor immune response including DCs was evaluated using the same PAN02 bilateral subcutaneous tumor model (Fig. 5A), intratumoral injection of Ad-p53 and OBP-702 significantly increased the recruitment of whole DCs (CD11b+/CD11c+/MHC-II+) and matured DCs (CD86+/CD11b+/CD11c+/MHC-II+) into draining lymph nodes, compared with OBP-301 (Fig. 5B). Immunohistochemical staining of tumors showed that OBP-702 intratumoral injection significantly increased the number of p53-positive and CD11c-positive cells in both treated and untreated tumors, resulting in significant recruitment of CD8-positive cells, whereas Ad-p53 increased these cells only in treated cells (Fig. 5C). The CTL assay showed that splenocytes collected from mice treated with Ad-p53 and OBP-702 showed significantly stronger cytotoxicity against PAN02 cells than OBP-301 (Fig. 5D, Table S1). These results suggested that intratumoral injection of OBP-702 had the potential to activate systemic antitumor immunity via DC stimulation as Exo702 did, and p53 may have been involved in this mechanism.

### Long-term antitumor effects of OBP-702 on PDAC tumors via systemic activation of antitumor immunity

**Fig. 6:**
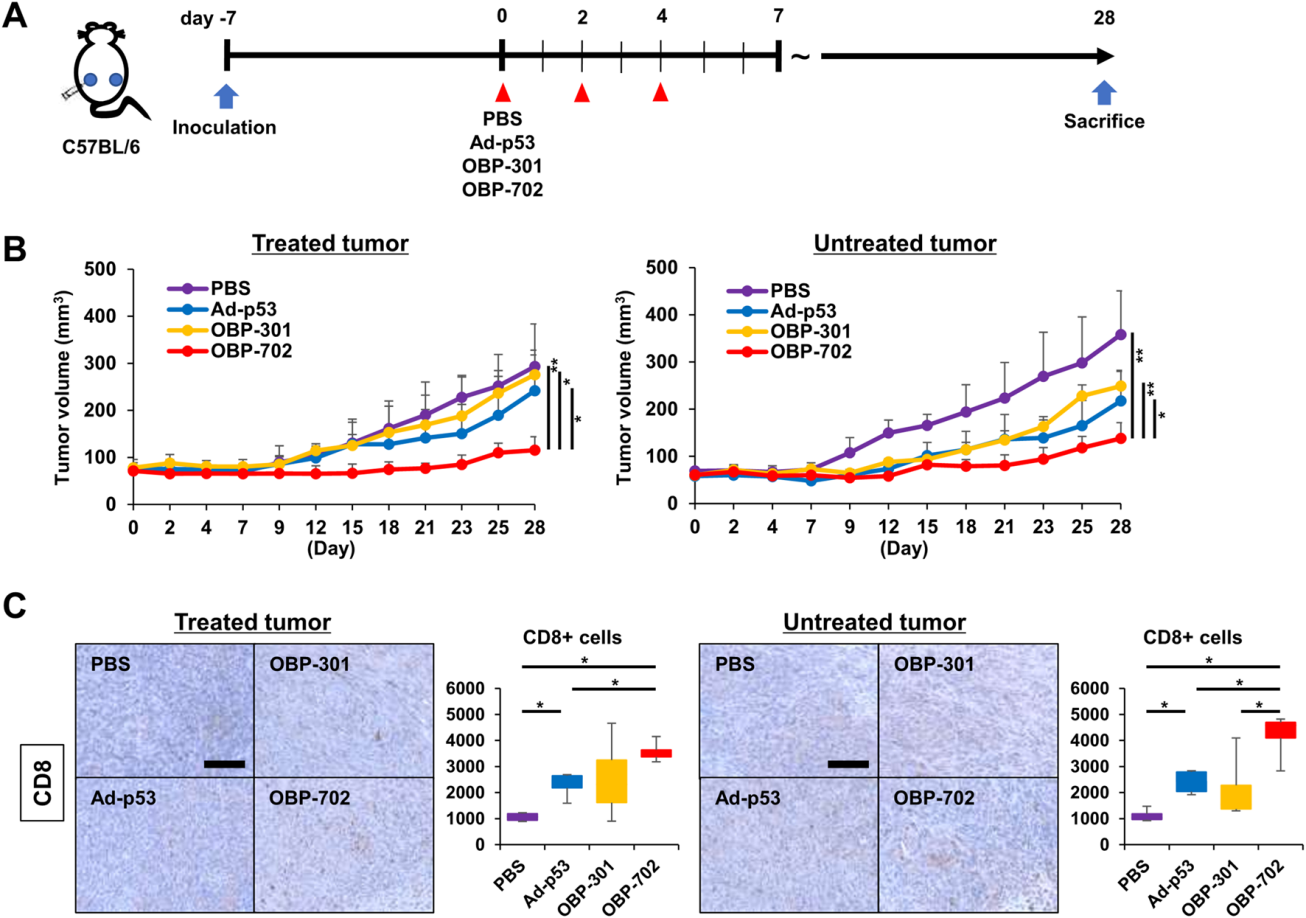
Long-term antitumor effects of OBP-702 on PDAC tumors via systemic activation of antitumor immunity **A** Study protocol. Briefly, in a bilateral PAN02 subcutaneous tumor model, one side was treated with Ad-p53, OBP-301, or OBP-702 intratumorally (1 × 10^8^ PFU) 3 times a week, and the other side was left untreated. Mice were sacrificed 28 days after treatment initiation for analysis (*n* = 4). **B** PAN02 tumor volumes of both sides were monitored separately until 28 days after treatment initiation (*n* = 4). *, *p* < 0.05. **, *p* < 0.01 (Student’s *t*-test). **C** Representative figures of immunohistochemical staining for CD8 in treated and untreated tumors harvested 28 days after treatment initiation are shown. Scale bar, 100 μm. Median numbers of CD8-positive cells from 5 selected fields were assessed statistically (*n* = 4). *, *p* < 0.05 (Wilcoxon signed-rank test)

Finally, when the long-term antitumor effects of OBP-702 were evaluated using the same PAN02 bilateral subcutaneous tumor model (Fig. 6A), intratumoral injection of OBP-702 significantly suppressed the tumor growth on both treated and untreated sides compared with Ad-p53 and OBP-301 28 days after treatment initiation (Fig. 6B, Fig. S7). Immunohistochemical staining of tumors showed that the facilitated recruitment of CD8-positive cells by OBP-702 was retained in both treated and untreated tumors 28 days after treatment initiation (Fig. 6C). These results suggested that intratumoral injection of OBP-702 produced long-lasting antitumor effects via durable activation of systemic antitumor immunity.

## Discussion

Oncolytic virus therapy is a novel treatment strategy for cancer that leverages the ability of genetically engineered viruses to selectively infect and kill tumor cells. This approach not only directly destroys cancer cells, but it also systemically activates antitumor immunity by promoting an immune response at the local injected site. In addition, local treatment with oncolytic viruses has the potential to induce the abscopal effect, in which immune-mediated destruction of cancer cells occurs at distant metastatic sites not directly exposed to the virus. This systemic immune activation involves the release of tumor antigens and danger signals, which help to stimulate and educate the immune system to recognize and attack metastatic tumors throughout the body. Thus, oncolytic virus therapy offers a twofold benefit: localized tumor destruction and the potential for widespread antitumor immune responses. We previously showed that OBP-301 produced the abscopal effect via systemic antitumor activation after ICD induction and the direct delivery of virus particles by tumor-derived exosomes [9, 27]. OBP-301 demonstrated its potential in the present study. However, OBP-702 exhibited stronger antitumor effects by activating systemic antitumor immunity through more robust DC maturation. The difference between OBP-301 and OBP-702 is the presence or absence of p53, which indicates that the human p53 expressed by OBP-702 functions in a mouse model using PAN02 cells, although it is unclear whether this function is full or partial, as has also been shown in our previous reports [14–16]. Although the TEM images in Fig. 2A might raise doubts about whether the viruses were merely attached to the exosomes rather than inside them, we demonstrated in a previous report that the virus particles were indeed located within the exosomes [27]. This finding is also supported by other reports [30, 31].

It is known that p53 not only serves as a crucial tumor suppressor through the induction of apoptosis, but it also plays a significant role as a modulator of the immune system [32]. One of its key functions in this regard is the maturation of DCs, which are essential for initiating and regulating immune responses. Mature DCs are responsible for presenting antigens to T cells, thereby stimulating and activating them. This interaction is particularly important for the activation of antitumor immunity, since the effective stimulation of T cells by DCs can lead to the recognition and destruction of cancer cells. Thus, p53’s effects extend beyond its direct tumor-suppressing activities to encompass critical immune-modulating functions. In a PAN02 bilateral subcutaneous tumor model (Fig. 5), although OBP-702 treatment significantly increased mature DCs in draining lymph nodes compared with OBP-301, Ad-p53 treatment also increased mature DCs to a similar level as OBP-702, proving the importance of p53 in DC maturation by OBP-702.

The present study focused on the effects on DCs via tumor-derived exosomes, which are considered important in intercellular communication and carry a variety of bioactive molecules, including proteins, lipids, and nucleic acids [33]. Experiments using GW4869, an inhibitor of exosome release, and anti-CD63, an antibody targeting an exosome marker, demonstrated that exosomes played significant roles in DC maturation induced by OBP-702. However, we did not identify any specific factors encapsulated in or bound to the exosomes that are responsible for this function, despite performing comprehensive liquid chromatography/mass spectrometry analyses. This suggests that further investigation is needed to elucidate the precise components and mechanisms through which exosomes contribute to DC maturation. Identifying the key molecules involved in DC maturation could provide new targets for therapeutic intervention and improve the effectiveness of existing treatments.

**Fig. 7:**
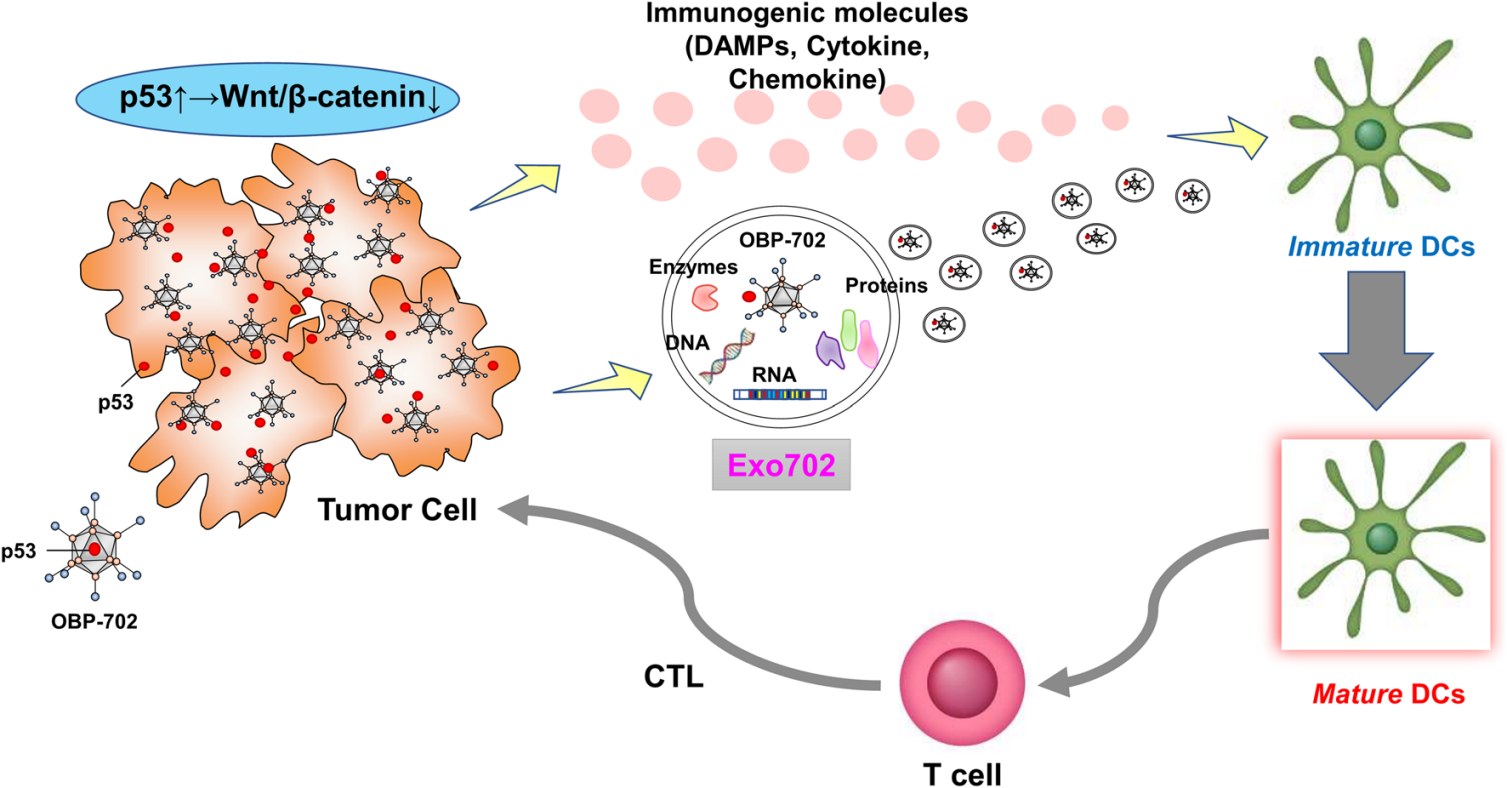
Schematic illustration

In the present study, DCs were shown to play a critical role, especially via tumor-derived exosomes, in the antitumor activity of OBP-702 (Fig. 7). Oncolytic virus therapy will be an attractive partner through the characteristics of enhanced antigen presentation, modulation of the tumor microenvironment, and stimulation of innate and adaptive immunity, in the further development of DC-based immunotherapy, such as DC vaccines.

## Electronic supplementary material

Below is the link to the electronic supplementary material.

Supplementary Material 1

## Data Availability

No datasets were generated or analysed during the current study.
