# Supplementary material for: Dendritic cell maturation is induced by p53-armed oncolytic adenovirus via tumor-derived exosomes enhancing systemic antitumor immunity
Source: Cancer Immunol Immunother. 2024 Nov 5;74(1):12. doi: 10.1007/s00262-024-03849-5 (PMC11538125; doi:10.1007/s00262-024-03849-5)
Supplement: Supplementary file 1 — Supplementary Material 1 [file 262_2024_3849_MOESM1_ESM.pdf]

Supplementary Information

Dendritic cell maturation is strongly induced by *p53*-armed oncolytic adenovirus via tumor-derived exosomes enhancing systemic antitumor immunity

Tomoko Ohtani[1], Shinji Kuroda[1,2], Nobuhiko Kanaya[1], Yoshihiko Kakiuchi[1,2], Kento Kumon[1], Masashi Hashimoto[1], Chiaki Yagi[1], Ryoma Sugimoto[1], Satoru Kikuchi[1], Shunsuke Kagawa[1,3], Hiroshi Tazawa[1,4], Yasuo Urata[5], Toshiyoshi Fujiwara[1]

[1] Department of Gastroenterological Surgery, Okayama University Graduate School of Medicine, Dentistry and Pharmaceutical Sciences, Okayama, Japan.

[2] Minimally Invasive Therapy Center, Okayama University Hospital, Okayama, Japan.

[3] Center for Clinical Oncology, Okayama University Hospital, Okayama, Japan.

[4] Center for Innovative Clinical Medicine, Okayama University Hospital, Okayama, Japan.

[5] Oncolys BioPharma, Inc., Tokyo, Japan.

Figure S1. Gene structures of Ad-p53, OBP-301 and OBP-702

Figure S2. Western blot analysis for exosomes

Figure S3. Cytotoxic assay using ExoCap on Panc-1 and MiaPaCa-2 cells

Figure S4. Representative figures of flow cytometry in Fig. 3A

Figure S5. Short-term antitumor effects of Exo702 on PAN02 tumors

Figure S6. Gating strategy of flow cytometry for mature DCs in draining lymph nodes

Figure S7. Long-term antitumor effects of OBP-702 on PAN02 tumors

Table S1. Absorbance values of the CTL assay shown in Fig. 5D

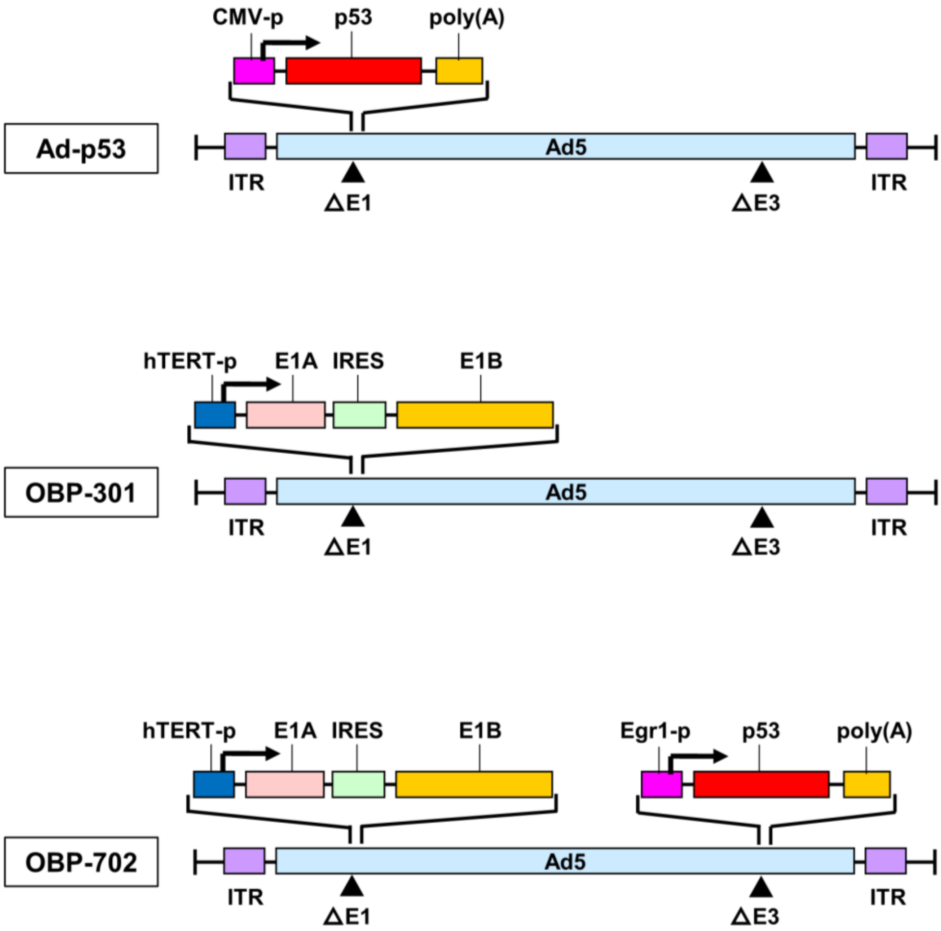

Figure S1. Gene structures of Ad-p53, OBP-301 and OBP-702

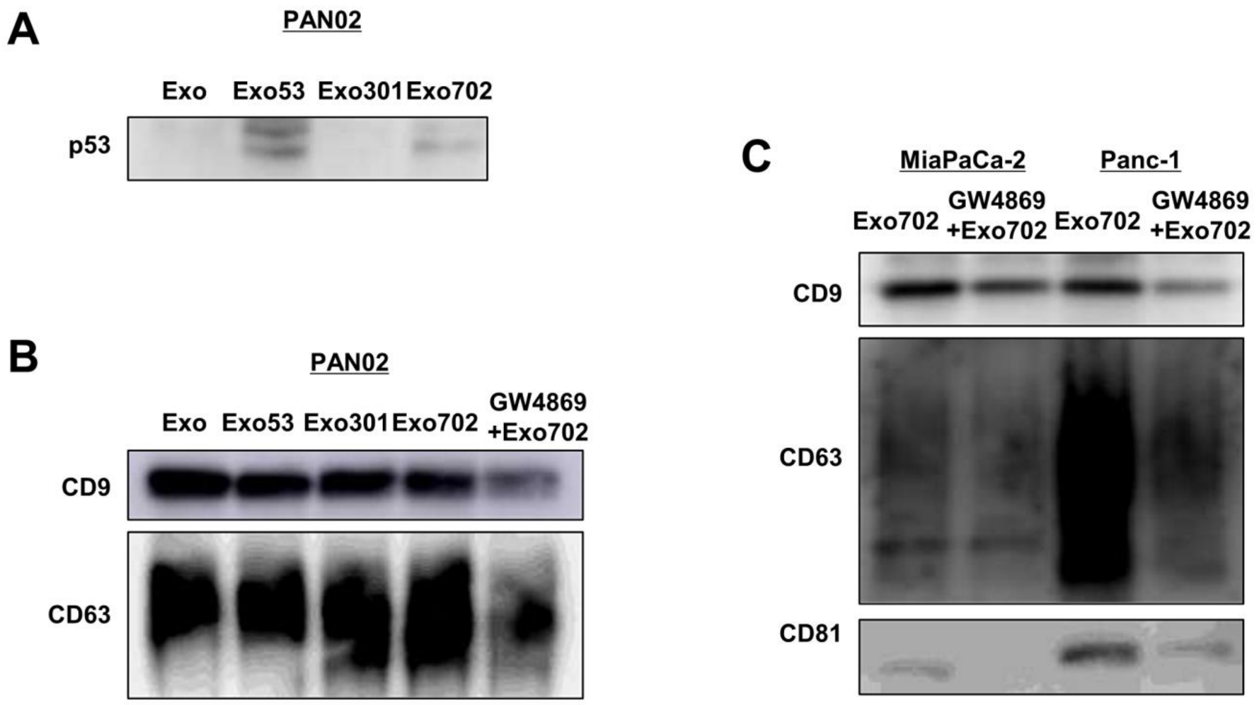

Figure S2. Western blot analysis for exosomes

(A) Lysates of Exo, Exo53, Exo301, and Exo702 derived from PAN02 cells were subjected to western blot analysis of p53. (B) Lysates of Exo, Exo53, Exo301, Exo702, and GW4869+Exo702 derived from PAN02 cells were subjected to western blot analysis of CD9 and CD63. (C) Lysates of Exo702 and GW4869+Exo702 derived from Panc-1 and MiaPaCa-2 cells were subjected to western blot analysis of CD9, CD63, and CD81.

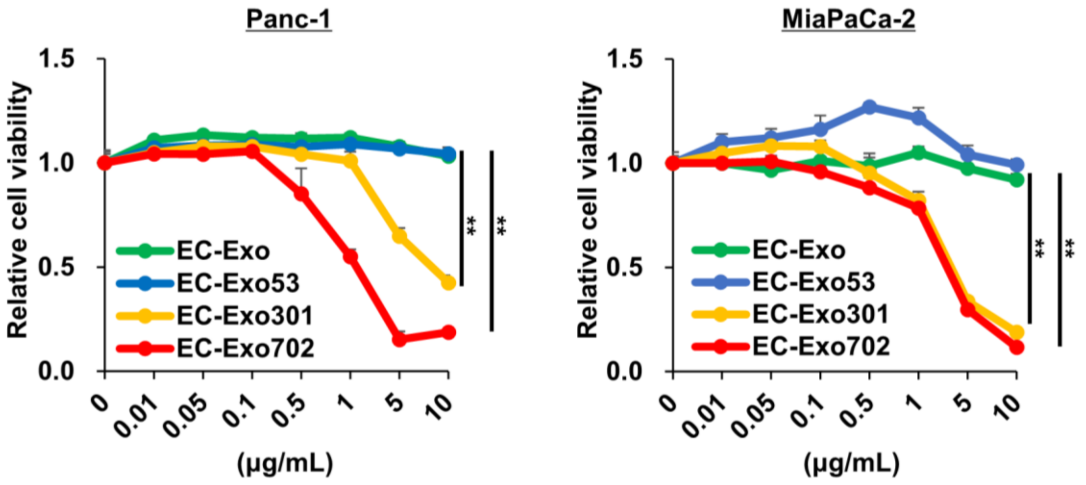

Figure S3. Cytotoxic assay using ExoCap (EC) on Panc-1 and MiaPaCa-2 cells

Viability of Panc-1 and MiaPaCa-2 cells was assessed using an XTT assay 3 days after treatment with EC-Exo, EC-Exo53, EC-Exo301, or EC-Exo702 at the indicated concentrations (n=5). EC was used to extract pure exosomes. **, $p < 0.01$ (Student's *t*-test).

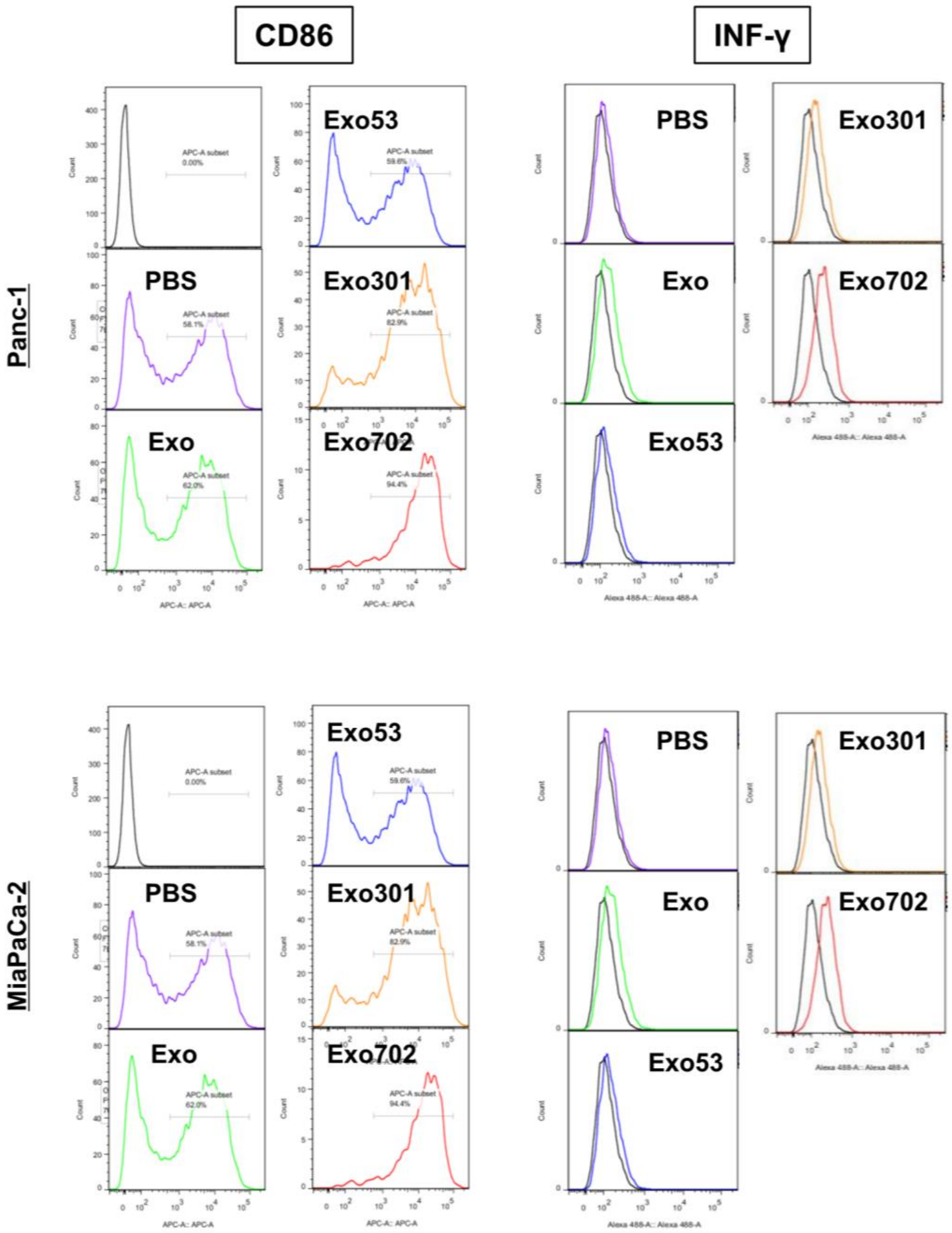

Figure S4. Representative figures of flow cytometry in Fig. 3A

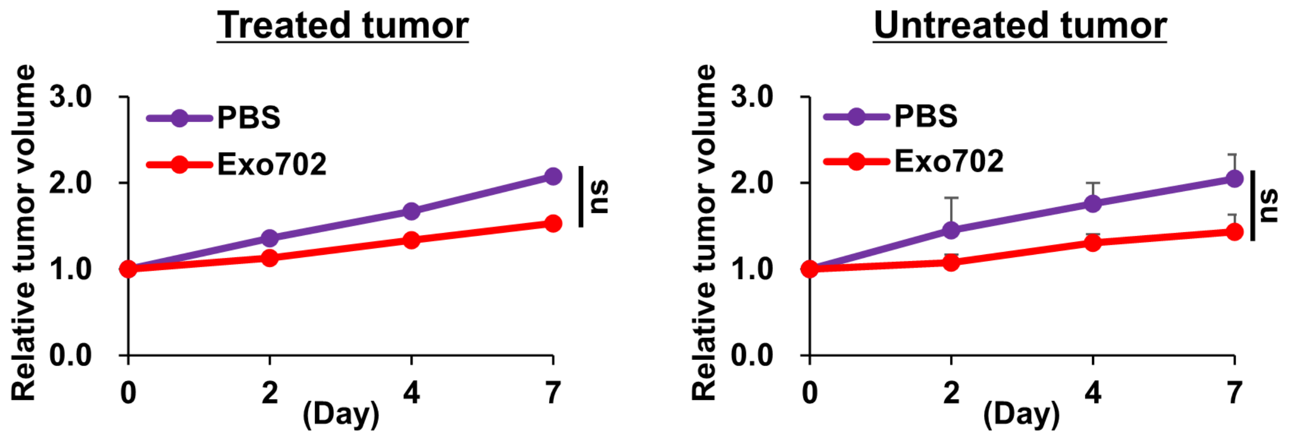

Figure S5. Short-term antitumor effects of Exo702 on PAN02 tumors

In a bilateral PAN02 subcutaneous tumor model, one side was treated with Exo702 intratumorally (20 μg) 3 times a week, and the other side was left untreated. PAN02 tumor volumes of both sides were monitored separately until 7 days after treatment initiation of Exo702 (n=3). Relative tumor volume to the baseline (day 0) was plotted using the same data as Fig. 4D. ns, not significant.

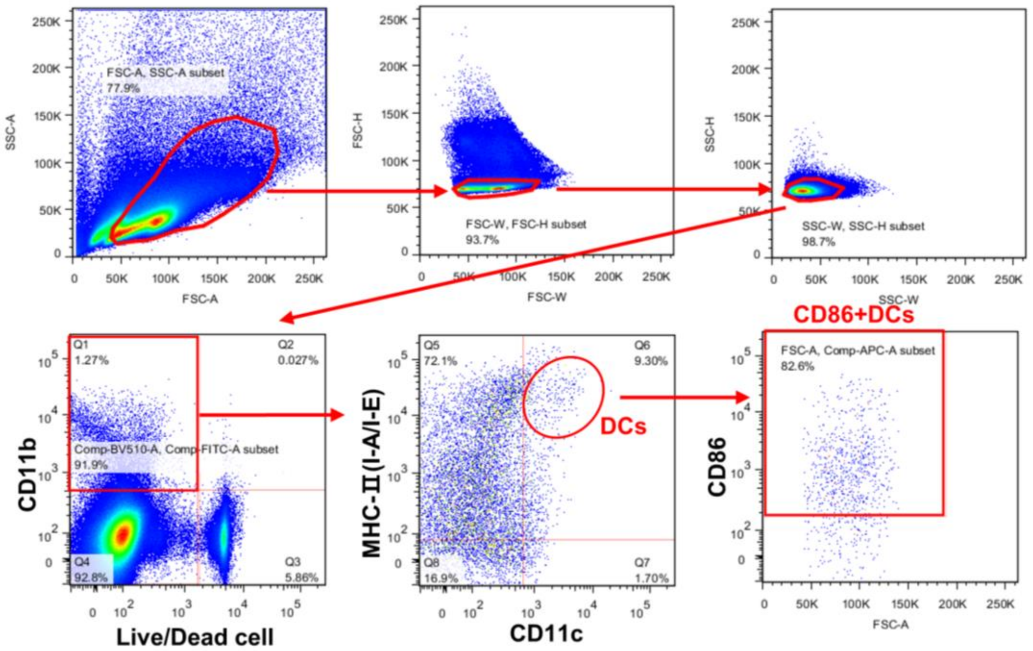

Figure S6. Gating strategy of flow cytometry for mature DCs in draining lymph nodes

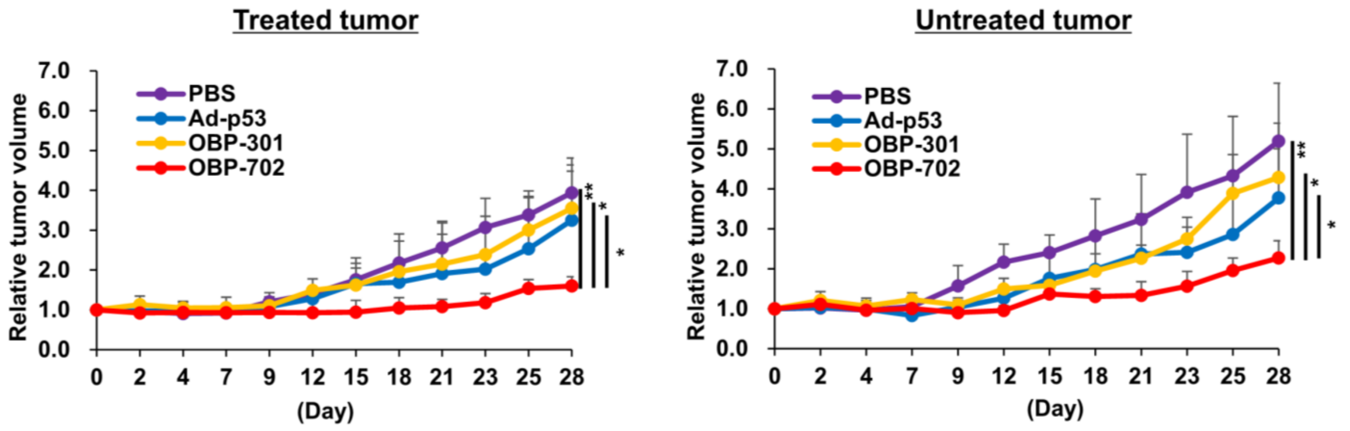

Figure S7. Long-term antitumor effects of OBP-702 on PAN02 tumors

In a bilateral PAN02 subcutaneous tumor model, one side was treated with Ad-p53, OBP-301, or OBP-702 intratumorally (1×108 PFU) 3 times a week, and the other side was left untreated. PAN02 tumor volumes of both sides were monitored separately until 28 days after treatment initiation (n=4). Relative tumor volume to the baseline (day 0) was plotted using the same data as Fig. 6B. *, $p < 0.05$. **, $p < 0.01$ (Student's *t*-test).

	1	2	3
PBS	0.2558	0.3764	0.3066
Ad-p53	0.4705	0.3739	0.3360
OBP-301	0.3244	0.3326	0.3129
OBP-702	0.3511	0.3617	0.3564
High control	0.8696	0.8783	0.8333
Low control	0.1775	0.2135	0.2040

Table S1. Absorbance values of the CTL assay shown in Fig. 5D

The cytotoxicity was calculated using the following formula:

Cytotoxicity (%) = ([effecter: target cell mix - effecter cell control] - low control) × 100 / high control - low control.

The values for PBS, Ad-p53, OBP-301, and OBP-702 correspond to (effecter: target cell mix - effecter cell control) in this formula.
